# Segmented assimilation trajectories of physician trust among internal migrants in Shanghai, China: A cross-sectional study

**DOI:** 10.1016/j.heliyon.2024.e37833

**Published:** 2024-09-18

**Authors:** Enhong Dong, Ting Xu, Yue Yan, Sheng Ji, Jiahua Shi, Haiwang Zhou, Yuping Liu, Cheng Huang, Baoshan Bu

**Affiliations:** a School of Nursing and Health Management, Shanghai University of Medicine & Health Science, Shanghai, 201318, China; b School of Media and Communication, Shanghai Jiao Tong University, Shanghai, 200240, Shanghai, China; c Institute of Healthy Yangtze River Delta, Shanghai Jiao Tong University, Shanghai, 200030, Shanghai, China; d School of Medicine, Tsinghua University, Beijing, 100084, China; e HuangPu District Health Promotion Center, Shanghai, 200040, China; f Institute of Urban and Demographic Studies, Shanghai Academy of Social Sciences, Shanghai, 200020, China; g School of Humanities and Management, Kunming Medical University, Kunming, 650500, China; h Antai College of Economics & Management, Shanghai Jiao Tong University, Shanghai, 200030, China; i Department of Logistics Service, Shanghai University of Medicine & Health Science, Shanghai, 201318, China; j The Center for Health Economics and Management, Shanghai Jiao Tong University, Shanghai, China

**Keywords:** Segmented assimilation, Physician trust, Internal migrant, China

## Abstract

Physician trust is necessary for improving physician–patient relationships and maintaining an effective health-care system. Most studies on the theory of segmentation assimilation have mainly focused on comparing immigrants in different countries and few studies have applied segmented assimilation to clarify physician–patient relationships, especially physician trust among internal migrants in the context of Chinese megacities. A random sample of 1200 internal migrants was collected through an online questionnaire conducted in Shanghai from August to December 2021. An exploratory K-means cluster analysis and multivariate logistic regression models were employed to identify patterns of segmented assimilation and examine their relationships with physician trust, as well as the factors influencing physician trust among internal migrants in Shanghai. Results show four main patterns were revealed, namely first-generation classic assimilation, first-generation integrative assimilation, first-generation segmentation, and second-generation underclass assimilation, supporting the theory of segmented assimilation. Association between assimilation pattern and physician trust was observed. A relationship was found between assimilation patterns and trust in physicians. Migrants belonging to the first-generation classic and integrative assimilation groups exhibited a higher level of trust in doctors compared to those in the segmented assimilation groups. Additionally, undergraduate and postgraduate education attainment, an annual income of <400 000 yuan, visiting the physician ≥2 times in the past year, and self-rated health status before and after population floating were significant contributing factors for good physician trust. The government can implement corresponding measures to maintain physician trust, improve cultural adaptability instead of only at the economic level among internal migrants, achieving the facilitation of their understanding of the health system and health service utilization to increase physician trust.

## 1 Introduction

### 1.1 Physician trust in China

Physician trust is a key measure of physician-patient relationships, and it is regarded as a collective interest that is necessary for improving physician-patient relationships and maintaining an effective healthcare system. However, a widespread concern is that patients’ trust in physicians is decreasing because of various threats to physician-patient relationships worldwide [1]. This phenomenon has also been observed in China, highlighting a broad trend that affects various countries. Since the

convening of the 19th National Congress of the Communist Party of China in 2017, physician-patient disputes have become a key social contradiction in China, particularly among internal migrants in megacities. This phenomenon is due to the considerable demand-supply gaps that exist in megacities. The disparity between the restricted availability of healthcare resources provided by government agencies and the urgent demand for health services among internal migrants has resulted in a divide between doctors and patients. Furthermore, because of restrictions on one's registered place of residence [2] and differences in medical insurance settlements resulting from urban-rural dualism in China [3], internal migrants always evaluate issues negatively and perceive discrimination. In addition, unmet needs related to medical services eventually lead to a poor evaluation of healthcare services, resulting in mutual mistrust between physicians and patients and tense physician-patient relationships. According to a report by the Shanghai Municipal Health Commission in 2018, more than 60 % of the medical disputes that occurred in Shanghai in 2017 involved internal migrants who were seeking medical treatment. In addition, only 46.4 % of the surveyed patients trusted physicians, and only 26.0 % of the surveyed physicians believed that their patients trusted them; these numbers were even smaller for migrant patients. Poor physician-patient relationships not only affect health service utilization but also cause potential problems for urban governance [4]. Therefore, physician-patient trust among internal migrants in megacities is a crucial topic for investigation in the context of population governance in China.

### 1.2 Immigrants’ segmented assimilation trajectories

In immigration assimilation research, two main assimilation theories exist: straight-line and segmented assimilation [5]. Straight-line assimilation proposes that each generation experiences greater upward mobility than the previous generation. Segmented assimilation, in contrast, outlines three possible outcomes for each generation: upward assimilation, downward assimilation, and upward mobility while maintaining a bicultural identity. It is the process by which immigrants are incorporated into various aspects and sectors of mainstream society such as education, employment, access to healthcare, and participation in civic life [6]. Three trends have been observed when analyzing the patterns of acculturation of immigrants entering a new culture: consonant acculturation, dissonant acculturation, and selective acculturation. These trends are based on the differences in acculturation between immigrants and second-generation immigrants, who may include young children accompanying immigrant parents or children born to immigrant parents. According to the theory of segmented assimilation, health-related segmented assimilation trajectories form when immigrants enter a host country or region; that is, they would maintain their health outcomes, which exhibit different segmentation characteristics relative to native residents, but are also gradually assimilated into the local population [7]. Portes and Zhou believed that during the process of segmentation and assimilation, immigrants exhibit three types of trajectories: upward assimilation (classic assimilation), downward assimilation (underclass assimilation), and selective assimilation (segmented assimilation) [6]. Upward assimilation indicates increasing acculturation into the middle class (e.g., embracing the prevailing societal norms and customs while discarding one's indigenous culture; path 1). Downward assimilation refers to integration into the underclass (e.g., economic hardship, limited educational levels, and preservation of the native cultural practices; path 2). Selective assimilation, often referred to as segmented acculturation, involves maintaining ethnic traditions while simultaneously advancing in economic status and educational attainment.; path 3). In addition to these three trajectories, scholars have proposed another derivative path, integrative assimilation (path 4). It is a type of classic assimilation with biculturalism that integrates an original culture with a host culture while preserving the advantages of education and income [8]. Several studies have asserted that the process of segmentation and assimilation refers to the adaptation and assimilation of cultural customs that have a major effect on health, in addition to various socioeconomic factors (e.g., diet/nutrition, stress experiences, support network maintenance, and substance abuse risk) [9]. The segmented assimilation theory can be applied to interpret how the adoption of values and behavioral involvement changes psychological well-being [10], social support networks [11], and behaviors of substance abuse risk among immigrants [12]. For example, Flórez and Abraído-Lanza argued that both second- and third-generation classic individuals tended to be more probable to be obese than those in the segmented pattern [13]. Ramírez discovered that the diet quality of a classic assimilation group was poorer than that of an underclass assimilated group, regardless of their educational and economic advantages, whereas the segmented and vulnerable groups exhibited higher or similar levels of dietary quality [14]. Castro et al. suggested that individuals in the upward assimilation category reported the greatest life contentment and the least frequent intake of unhealthy foods in comparison to other groups [12]. With regard to the doctor-patient relationship, the literature applying the segmented assimilation theory to study physician trust and patient satisfaction, especially among domestic migrants within the Chinese context, is lacking, while most previous studies on the segmented assimilation theory have mainly focused on inter-country immigration's diet-related diseases, including a higher risk of obesity, poorer diet and nutrition, and less favorable health outcomes [13,14].

### 1.3 Physician trust and acculturation

Because segmented assimilation examines how immigrants are incorporated into the dominant culture, acculturation is a key element. Based on our study team's literature review, though the literature exploring the links between acculturation processes and issues such as obesity, social strain, psychological health, and overall life satisfaction is relatively well-developed [[15], [16]], the research examining the relationship between acculturation and physician trust is sparse. Tarn asserted that the process of acculturation had a significant correlation with the level of trust patients placed in their healthcare providers, particularly among Japanese Americans and Japanese individuals. Furthermore, Tarn addressed that individuals who were more acculturated to Western customs and those whose physicians shared their ethnic background were more inclined to exhibit higher levels of trust, as the challenges posed by language and cultural differences were less pronounced [17]. Kim discovered that as immigrants became more acculturated after moving, their reported trust in healthcare providers grew [18]. Smith provided evidence that lower acculturation among adult caregivers of Hispanic origin for children participating in North Carolina's Medicaid program explains lower trust in healthcare providers among Hispanics compared to non-Hispanic blacks [19]. Zambrana recognized that English proficiency and the capability of providers to communicate in Spanish are significant elements contributing to racial and ethnic differences in access to and responsiveness from healthcare providers [20]. In addition, lifestyle integration (an adaptation to the behaviors, values, customs, and language of immigrants newly coming to a host country or region) was positively associated with physician trust [21]. In short, most studies on the theory of segmentation assimilation have mainly focused on comparing immigrants in different countries, and few studies have applied segmented assimilation to clarify physician-patient relationships, especially physician trust among internal migrants in the context of Chinese megacities. In doing so, the present study aims to utilize the theory of segmented assimilation as a foundational framework to conceptualize and measure acculturation, explore its relationship with trust in physicians among internal migrants in China, and identify the factors that affect this trust.

### 1.4 Literature review and hypotheses we proposed

Numerous scholars have researched and validated Berry's two-dimensional, 4-g acculturation strategies of assimilation, separation, integration, and marginalization. For example, Flannery conducted an empirical comparison among unidirectional, bidirectional, and tridirectional acculturation Models and proposed four types of acculturation categories: traditional or separated (High African-Low Australia), assimilated (High Australian-Low African), integrated or bicultural (High on both), and marginalized (low on both) [22]. Lee studied Korean-American acculturation using a two-dimensional scale developed by Berry and identified three acculturation strategies: integration, assimilation, and separation [23]. Renzaho classified African migrant children aged 3–12 years in Australia into four categories: traditional, integrated, assimilated, and marginalized [24]. Miao and Xiao categorized the incoming migrant population in eight cities in China as culturally adapted: integrated, assimilated, separated, and marginalized groups, and compared each group with the corresponding self-reported health status and mental health between groups [25]. Therefore, we propose the following hypothesis.

H1 Internal migrants in Shanghai exhibit various patterns of assimilation that support the theory of segmented assimilation.

Tatarko and Rodionov found any of the three types of trust generalized, social, and institutional trust were associated with assimilation-oriented acculturation expectations, where only social trust showed a positive link with "integration" acculturation, whereas institutional and generalized trust did not exhibit such a connection. Moreover, no adverse associations were found between the different forms of trust and the acculturation expectations of "segregation" or "exclusion" [26]. The blending of cultural backgrounds has been demonstrated as the optimal adaptation, yielding the most favorable health results and psychological well-being [27,28] for immigrants. Matera reported that Muslim immigrants who were perceived as abandoning their ancestral cultural practices (assimilation acculturation) elicited more favorable subjective responses than those perceived as maintaining their heritage culture (segmented acculturation) [29]. Lajunen and Wróbel suggested that the acculturation process, marked by either assimilation or integration, results in an increased level of trust in health authorities among Polish immigrants. Furthermore, first-generation immigrants often perceive the institutions of their new country as superior to those in their homeland [30]. Therefore, we propose the following two hypotheses.

H2a First-generation classic assimilated migrant groups exhibit a higher level of physician trust relative to first-generation segmented groups.

H2b First-generation integrative assimilated groups exhibit a higher level of physician trust relative to first-generation segmented groups.

Immigrants and refugees undergo a process of downward mobility, also known as downward assimilation. This is especially the case for recent arrivals who previously held professional positions in their home countries but now find themselves in roles such as technicians. Individuals of lower social standing may also encounter this phenomenon. Previous studies have indicated that for certain immigrants and refugees, even though downward mobility might be intended to be a temporary phase, they could potentially endure it throughout their lives, potentially passing on the associated hardships to their offspring, which may lead to a decline in their children's generation, adopt marginalization-type acculturative coping strategies, and be excluded from mainstream society [31]. Research has demonstrated that marginalization exerts a substantial adverse impact on psychological adjustment and overall well-being. Kosic discovered that among Polish immigrants in Italy, marginalization correlated with diminished sociocultural and psychological adaptation [32]. Research conducted by Sam and Berry on young immigrants in Norway found that there is a positive correlation between acculturative stress and marginalization [33]. Moreover, some scholars have reported that marginalized acculturation negatively affects trust in health systems or authorities [30]. Therefore, we propose the following hypothesis.

H2c Second-generation underclass assimilated groups exhibit a lower level of physician trust relative to first-generation segmented groups.

## 2 Data and methods

### 2.1 Data collection

The research team collected data from 1200 internal migrants in Shanghai between January 2021 and December 2021 using an online questionnaire survey (Questionnaire Star Platform). A systematic sampling technique was employed to choose qualified participants. Individuals were classified as internal migrants and included in the research if they met the following criteria: (1) they were 18 years of age or older, and (2) they were not originally from Shanghai but had received legal permanent or temporary residency permits from the Migrant Population Management Office for a minimum of 6 months.

An online questionnaire was completed using a single IP address. A questionnaire response was excluded if it (1) was completed in <200 s (15, 1.25 %) (2) exhibited signs of careless responding (e.g., the same response was used for multiple questions), (45, 3.75 %), (3) contained ≥5 % missing values (10, 0.83 %), (4) or contained logic errors (19,1.57 %). Finally, 1117 eligible participants were included (response rate: 92.6 %).

This study was carried out in accordance with the Shanghai University of Medicine and Health Sciences Institutional Review Board for the Protection of Human Subjects on March 5, 2019 (No. 2019-gskyb-02-372424198012222511). All the participants provided written informed consent.

### 2.2 Methods

#### 2.2.1 Measures

Physician trust was measured using the Chinese version of the Wake Forest Physician Trust Scale that was improved by the research team [34,35]. The scale contains 12 items covering four dimensions: environmental, interpersonal, technical, and management quality trust. Each item was rated on a 5-point Likert-type scale, with endpoints ranging from 1 (*completely disagree*) to 5 (*fully agree*) points. A higher mean score for the 12 items indicated higher physician trust. For convenience, the average trust score was dichotomized into *good* and *bad* scores. The internal consistency coefficients for environmental quality trust, interpersonal quality trust, technical quality trust, and management quality trust were 0.764, 0.831, 0.734, and 0.832, respectively, and the reliability of the total scale was 0.876.

Previous research has examined various metrics of health service usage, such as the frequency of hospital admissions, doctor appointments, emergency room visits, and physical examinations. [36,37], we measured health service utilization using two questions, in the study, namely “How frequently did you undergo physical examinations in the past year?” “How frequently did you see a doctor in the past year?” Additionally, as health service utilization is also determined by healthcare-seeking behaviors [38,39], we added another question to indirectly assess how health services are used: “In the past year, what has been your first-choice medical institution when you need to see a physician?” The first two questions are rated from 0 (no) to 3 (3 times or more) points, with a higher score indicating greater utilization of health services. For the question on medical institution preference, institutions were mainly classified into “hospitals” and “primary health service institutions.”

10.13039/100018696Health status was assessed based on the self-rated health (SRH) status scale developed by the research team [40], which consists of five items that cover physical health, mental health, and social support. Each item is rated from 1 (*very good*) to 5 (*very poor*). A lower mean score for the five items indicates a more favorable health status. For convenience, the mean score was dichotomized into two categories (: *good* and *poor*). The coefficient of internal consistency for the overall health status measure was 0.819.

The number of type I and type II diseases that a respondent had was measured using a single question. Type I diseases include a total of 10 chronic conditions, which are asthma, back pain, hypertension, high cholesterol, diabetes, allergies, migraines, ulcers, bronchitis, and arthritis. The responses were rated from 1 to 3 (1 point, *no disease*; 2 points, *one disease*; and 3 points, *two or more diseases*). Type II diseases comprised three critical diseases (: heart disease, cancer, and blood disease). Responses were rated from 1 to 2 points (1 point, *no disease*; 2 points, *one or more disease*). The classifications and measurements of the main variables of interest are presented in Table S1.

#### 2.2.2 Analysis method

Initially, a K-S test was applied to assess the data's adherence to a normal distribution. This was succeeded by descriptive statistics, one-way ANOVAs, and independent t-tests. Covariates that showed statistical significance in the one-way ANOVAs and t-tests were then selected for inclusion in subsequent multivariate logistic regression analyses.

An explorative K-mean cluster analysis was conducted to explore if distinct assimilation patterns, as hypothesized by the segmented assimilation theory, could be identified from the gathered data. Prior to the analysis, all variables were normalized to mitigate the impact of disparities in standard deviations or means. Given the diverse scales of the measures, continuous variables were converted into z-scores (mean = 0; standard deviation = 1). The selection of variables was guided by the SA framework, emphasizing socioeconomic factors (such as education status and income), acculturation processes, proficiency in the Shanghainese dialect, and generational status as key elements of the theorized assimilation patterns.

Notably, for acculturation, we examined the respondents' relationships with their host and original cultures by referring to the study by Miao and Xiao [25]. Their relationship with their host culture (adaptation) was measured by asking respondents if they agreed with four statements, for example,” Compared with local citizens, my hygiene habits are different.” The Cronbach's α of the four items was 0.87. Responses were recorded as 1 (*strongly agree*), 2 (*agree*), 3 (*neither agree nor disagree*), 4 (*disagree*), or 5 (*strongly disagree*), with a higher value indicating a greater level of adoption. The maintenance of one's original culture was measured by asking a respondent if they agreed with the four statements, for example, “It is important for me to follow my hometown customs (e.g., marriage- and funeral-related customs)”. The respondents' responses, which ranged from *strongly disagree* to *strongly agree*, were recoded as values between 1 and 5, with higher values indicating a higher level of maintenance of the original culture. The Cronbach's α of the four items was 0.80.

The proficiency in the Shanghainese dialect was evaluated through two questions where participants were asked to rate their speaking and listening skills on a 4-point Likert scale, with endpoints ranging from 1 (*very well*) to 4 (*not at all*). For generational status, the respondents were classified into first-, second-, and third-generation migrants based on their places of birth and those of their parents [13]. The respondents were classified as first-generation migrants if they were born outside Shanghai, second-generation migrants if they were born in Shanghai to at least 1 city-born parent, and third-generation migrants if they were born in Shanghai to two Shanghai-born parents.

Age [41], sex [42], marital status, education level, income [43], health service utilization [44], and the number of type-І and type-Ⅱ diseases [18,40] are all associated with physician trust; in the present study, these variables were included as covariate factors and adjusted for the logistic regression model used in the present study.

We constructed Models 1, 2, 3, and 4 to examine the salient effect of the segmented assimilation theory. Model 1 was an unadjusted null model, Model 2 was Model 1 adjusted for the variables of demographic and socioeconomic characteristics, Model 3 was Model 2 adjusted for health service utilization, and Model 4 was Model 3 with the addition of the variables of SRH status before and after coming to Shanghai and the number of type I and type II diseases.

The variance inflation factor (VIF) was employed to detect multicollinearity among the predictor variables. Variables with a VIF value less than 5 were considered to have an acceptable level of multicollinearity and were thus included in the multivariate regression analysis. Subsequently, to assess the model's fit and its predictive power, Receiver Operating Characteristic (ROC) curves and the Area Under the Curve (AUC) metric were utilized.

### 2.3 Statistical software

All analyses were performed with Stata 15.0 (StataCorp LP, College Station, TX, USA). Statistical significance was assessed using a two-tailed test, with a significance level set at P < 0.05.

## 3 Results

### 3.1 Respondent characteristics and various patterns of segmented assimilation

Among the 1117 respondents who submitted valid responses, 85.5 % were aged 18–39 years, 51.8 % (579) were male, 74.8 % (836) were married, and 62.9 % (703) had completed university or junior college education, 62.1 % (690) were covered by urban employee medical insurance, and 46.3 % (517) had an annual income of 110 000–250 000 yuan (Table 1).

**Table 1:** Results of the descriptive and univariate analysis (n = 1117).

Variables	n(%)	Physician trust		χ^2^/t/P values
		Good	Bad	
		N1 (%)	N2(%)	
**Segmented assimilation patterns**(**n = 1111**)				χ^2^ = 27.474 P < 0.001
First-generation classic assimilation	210(18.8)	173(82.4)	37(17.6)	
First-generation integrative assimilation	301(26.9)	263(87.4)	38(12.6)	
First-generation segmented	176(15.8)	127(72.2)	49(27.8)	
Second-generation underclass assimilation	424(38)	310(73.1)	114(26.9)	
**Years of residency**(**n = 1110**)				χ^2^ = 40.3915 p = 0.022
**<1 year**	187(16.7)	140(74.9)	47(25.1)	
1–5 years	465(41.6)	378(81.3)	87(18.7)	
5–10 years	241(21.6)	184(76.3)	57(23.7)	
≥10 years	217(19.4)	168(77.4)	49(22.6)	
**Age** (**n = 1113**)				t = 1.6566 p = 0.0979
≤39 old years	955(85.5)	757(79.3)	198(20.7)	
40-60 old years	158(14.1)	116(73.4)	42(26.6)	
**Gender (n = 1114**)				t = −0.1837 p = 0.870
Male	579(51.8)	453(78.2)	126(21.8)	
Female	535(47.9)	421(78.7)	114(21.3)	
**Marital status**(**n = 1116**)				t = −3.1153 p = 0.001
No	280(25.1)	200(71.4)	80(28.6)	
Yes	836(74.8)	676(80.9)	160(19.1)	
**Educational level (n = 1117)**				χ^2^ = 1.1395 p = 0.768
High school or below	96(8.6)	76(79.2)	20(20.8)	
University (including junior college)	157(14.1)	121(77.1)	35(22.9)	
Graduate or above	703(62.9)	557(79.2)	146(20.8)	
	161(14.4)	122(75.8)	39(24.2)	
**Profession type (n = 1116**)				χ^2^ = 16.3530 p = 0.003
unemployment(or retired)	97(8.7)	68(70.1)	29(29.9)	
Civil servant	86(7.7)	56(65.1)	30(34.9)	
Company (or enterprise)	735(65.9)	595(81.0)	140(19.1)	
Migrant workers	82(7.4)	67(81.7)	15(18.3)	
Skilled workers	116(10.4)	90(77.6)	26(22.4)	
**Place of birth**(**n = 1114**)				χ^2^ = 4.6213 p = 0.363
Shanghai	112(10)	94(83.9)	18(16.1)	
Eastern China (excluding Shanghai)	558(50)	442(79.2)	116(20.8)	
Central China	264(23.6)	203(76.9)	61(23.1)	
Western China	123(11)	90(73.2)	33(26.8)	
North East china	57(5.1)	44(77.2)	13(22.8)	
**Annual income (N = 1111)**				χ^2^ = 14.3033 p = 0.002
CNY100,000 or below	288(25.8)	204(70.8)	84(29.2)	
CNY110,000 -CNY250,000	517(46.3)	423(81.8)	94(18.2)	
CNY260,000 -CNY400,000	222(19.9)	178(80.2)	44(19.8)	
CNY410,000 -CNY600,000	55(4.9)	44(80)	11(20)	
CNY600,000 or more	30(2.7)	25(83.3)	5(16.7)	
**Insured type**(**n = 1112**)				χ^2^ = 8.0746 p = 0.089
UEBMI	690(62.1)	542(78.6)	148(21.4)	
URRMI	179(16.1)	148(82.7)	31(17.3)	
FI	41(3.7)	31(75.6)	10(24.4)	
CMI or others	141(12.6)	111(78.7)	30(21.3)	
No insured	61(5.5)	40(65.6)	21(34.4)	
**The frequency of physician visits physician in the past year**(**n = 1093**)				χ^2^ = 9.6600 p = 0.044
None	237(21.2)	173(73)	64(27)	
Once	367(32.9)	290(79)	77(21)	
Two time	305(27.3)	255(83.6)	50(16.4)	
Three times or more	184(16.5)	140(76.1)	44(23.9)	
**The frequency of physical examinations in the past year**(**n = 1101**)				χ^2^ = 6.6410 p = 0.084
None	247(22.1)	185(74.9)	62(25.1)	
Once	616(55.1)	477(77.4)	139(22.6)	
Two time	183(16.4)	153(83.6)	30(16.4)	
Three times or more	55(4.9)	47(85.5)	8(14.5)	
**The preferred health-care institution**(**n = 924**)				t = −0.1923 p = 0.847
Hospitals	698(62.5)	558(79.9)	140(20.1)	
Primary care institutions	226(20.2)	182(80.5)	44(19.5)	
**The number of type-І disease**^**a**^(n = 1113)				χ2 = 3.7138 p = 0.156
None of above	698(62.7)	537(76.9)	161(23.1)	
One	342(30.7)	280(81.9)	62(18.1)	
Two or more	73(6.6)	55(75.3)	18(24.7)	
**The number of type-Ⅱ disease**^**b**^(n = 1108)				t = 1.3242 p = 0.1857
None of above	1036(93.5)	817(78.9)	219(21.1)	
One or more	72(6.5)	52(72.2)	20(27.8)	
**SRH before floating**(**n = 1115**)				t = −3.6407 p < 0.001
Favorable	1060(95.1)	844(79.6)	216(20.4)	
Not favorable	55(4.9)	30(54.6)	25(45.5)	
**SRH after floating**(**n = 1117**)				t = −11.1487 p < 0.001
Favorable	902(80.8)	777(86.1)	125(13.9)	
Not favorable	215(19.2)	99(46.1)	116(53.9)	

a: type- І disease includes 10 common chronic health conditions, namely asthma, back pain, hypertension, hyperlipidemia, diabetes, allergies, migraine, ulcers, bronchial inflammation, and arthrit; b:type- II disease encompassed three common critical diseases: heart disease, blood-related cancers (e.g., leukemia), and other cancers.SRH:self-rated health; UEBMI: urban employee basic medical insurance; URRBMI: urban and rural residents basic medical insurance; FI:free insurance; CMI: Commercial medical insurance.

After K-means clustering analysis was performed, four main patterns were revealed: first-generation classic assimilation, first-generation integrative assimilation, first-generation segmentation, and second-generation underclass assimilation. The distribution of the clustering variables for each group is presented in Table S2.

### 3.2 Univariate analysis and correlation analyses

By conducting S-K normality testing, the data for all the dependent and independent variables of interest in this study were examined to be acceptable for subsequent analyses; specifically, their skewness values approached zero and their kurtosis values were all <3.

Through comparisons of the means of the various variables of interest, the variables significantly associated with physician trust were revealed to be segmentation assimilation type (χ^2^ = 27.47, *P* < 0.001), YOR (χ^2^ = 40.39, *P* = 0.002), age (*t* = 1.6566, *P* = 0.0979), marital status (*t* = −3.115, *P* = 0.001), occupation (χ^2^ = 16.35, *P* = 0.003), income (χ^2^ = 14.303, *P* = 0.002), medical insurance type (χ^2^ = 8.07, *P* = 0.089), number of physician visits in the past year (χ^2^ = 9.66, *P* = 0.044), number of physical examinations in the past year (χ^2^ = 6.64, *P* = 0.084), self-reported health before population floating into Shanghai (*t* = −3.64, *P* < 0.001), and self-reported health after floating (*t* = −11.149, *P* < 0.001) (Table 1).

### 3.3 Multivariate logistic regression analysis

Through multicollinearity testing, the VIFs of the independent variables used in the logistic regression model were found to be between 1.06 and 1.45, indicating the absence of multicollinearity.

The results of Model 1 revealed that the odds ratio (OR) values of first-generation classic assimilation patterns and first-generation integrative assimilation patterns were 1.804 (*P* < 0.01) and 2.670 (*P* < 0.001), respectively, indicating that, relative to respondents in the first-generation segmentation group, those who exhibited first-generation classic assimilation patterns and first-generation integrative assimilation patterns were 1.804 and 2.670 times more likely to report a high level of physician trust. However, the OR for the second-generation underclass was insignificant.

When the covariates of YOR, age, marital status, occupation, income, medical insurance type, number of physician visits and physical examinations in the past year, and self-reported health before and after floating were controlled for, the association between assimilation patterns and physician trust initially increased (i.e., Models 2 and 3), but subsequently decreased (i.e., Model 4). In Model 4, the OR values of the respondents in the first-generation classic and integrative assimilation groups were 1.320 (*P* < 0.05) and 1.988 (*P* < 0.01), respectively. That is, respondents in the first-generation assimilation groups were 1.320–1.988 times more likely to report a high level of physician trust than those in the first-generation segmented assimilation group.

Regarding the covariates that could influence physician trust, the respondents who completed university education and those who completed graduate or higher education were 53.7 % and 57.2 %, respectively, less likely to express a high degree of trust in physicians compared to those who completed junior high school education or below (OR = 0.467, *P* < 0.05; OR = 0.428, *P* < 0.05). By contrast, the respondents who had annual incomes of 110 000–250 000 yuan and 260 000–400 000 yuan were 1.917 and 1.800 times, respectively, more likely to indicate a high level of trust in physicians compared to individuals with an annual income of 100 000 yuan or less (OR = 1.917, *P* < 0.001; OR = 1.800, *P* < 0.05). Relative to respondents who did not see a physician, those who consulted a physician two or more times were 2.057 times more likely to express a high level of trust in physicians (OR = 2.057, *P* < 0.001). In a similar vein, individuals who indicated they had good health both prior to and after moving to the city were 2.282 and 6.361 times more likely, respectively, to express a high level of trust in their physicians compared to those who reported poor health (OR = 2.282, P < 0.001; OR = 6.361, P < 0.001). In Model 4 (Table 2), the covariates YOR, age, marital status, occupation, type of medical insurance, and number of physical examinations in the past year were not significant.

**Table 2:** Results of multivariate logistic regression analysis (n = 1117).

Independent variables	Model1^a^	Model2^b^	Model3^c^	Model4^d^
	OR (CI)	OR (CI)	OR (CI)	OR (CI)
**Segmented assimilation patterns**				
**First-generation segmented**	1.000	1.000	1.000	1.000
**First-generation classic assimilation**	1.804∗∗	1.932∗∗	1.951∗∗	1.320∗
	(1.111–2.929)	(1.262–2.956)	(1.262–3.018)	(1.152–2.020)
**First-generation integrative assimilation**	2.670∗∗∗	2.692∗∗∗	2.709∗∗∗	1.988∗∗
	(1.663–44.290)	(1.783–4.066)	(1.776–4.132)	(1.152–3.430)
second-generation underclass assimilation	1.050	1.165	1.224	1.083
	(0.708–1.556)	(0.824–1.645)	(0.857–1.748)	(0.737–1.594)
**Years of residency**				
<1 year		1.000	1.000	1.000
1–5 years		1.168	1.203	1.501
		(0.817–1.671)	(0.832–1.740)	(0.930–2.422)
5–10 years		0.763	0.755	0.766
		(0.505–1.154)	(0.495–1.153)	(0.449–1.307)
≥10 years		0.961	1.022	1.389
		(0.614–1.504)	(0.645–1.620)	(0.770–2.508)
**Age**				
≤39 old years		1.000	1.000	1.000
40-60 old years		0.622∗∗	0.607∗∗	0.686
		(0.420–0.921)	(0.407–0.906)	(0.413–1.142)
**Gender**				
Male		1.000	1.000	1.000
Female		0.941	0.965	1.051
		(0.725–1.223)	(0.736–1.266)	(0.740–1.493)
**Marital status**				
No		1.000	1.000	1.000
Yes		1.689∗∗∗	1.556∗∗	1.006
		(1.235–2.311)	(1.129–2.145)	(0.654–1.548)
**Profession type**				
Unemployment (or retired)		1.000	1.000	1.000
Civil servant		0.649	0.702	0.654
		(0.353–1.191)	(0.376–1.310)	(0.289–1.482)
Company (or enterprise)		1.157	1.167	0.875
		(0.721–1.856)	(0.714–1.906)	(0. 474–1.614)
Migrant workers		1.523	1.589	1.412
		(0.816–2.844)	(0.843–2.995)	(0.705–2.828)
Skilled workers		0.977	1.118	0.846
		(0.553–1.727)	(0.609–2.053)	(0.446–1.605)
**Insured type**				
No insured		1.000	1.000	1.000
UEBMI		1.390	1.698	1.958
		(0.730–2.646)	(0.859–3.353)	(0.895–4.282)
URRMI		1.873	2.048	2.213
		(0.985–3.563)	(0.031–4.069)	(0.004–4.877)
FI		1.520	1.612	1.714
		(0.630–3.669)	(0.636–3.083)	(0.639–4.596)
CMI or others		1.555	1.791	2.344
		(0.795–3.041)	(0.878–3.652)	(0.839–3.290)
**Educational level**				
**Junior or below**		1.000	1.000	1.000
**High school**		0.814	1.141	0.885
		(0.442–1.501)	(0.597–2.181)	(0.448–1.745)
**University (including junior college)**		0.515∗	0.510∗	0.467∗
		(0.281–0.944)	(0.377–0.968)	(0.233–0.938)
Graduate or above		0.474∗	0.455∗	0.428∗
		(0.233–0.964)	(0.322–0.855)	(0.186–0.983)
**Annual income**				
CNY100,000 or below		1.000	1.000	1.000
CNY110,000 -CNY250,000		1.959∗∗∗	1.917∗∗∗	1.917∗∗∗
		(1.356–2.829)	(1.312–2.799)	(1.277–2.877)
CNY260,000 -CNY400,000		1.792∗∗	1.618∗	1.800∗
		(1.150–2.793)	(1.016–2.576)	(1.088–2.976)
CNY410,000 -CNY600,000		1.828	1.488	1.600
		(0.944–3.540)	(0.737–3.006)	(0.753–3.403)
CNY600,000 or more		2.122	2.022	2.192
		(0.824–5.462)	(0.750–5.450)	(0.796–6.037)
**The frequency of physical examinations in the past year**				
None			1.000	1.000
Once			0.771	0.766
			(0.533–1.113)	(0.522–1.123)
Two time			1.152	1.043
			(0.710–1.870)	(0.611–1.781)
Three times or more			1.194	0.945
			(0.556–2.567)	(0.429–2.081)
**The frequency of physical visits in the past year**				
None			1.000	1.000
Once			1.315	1.509
			(0.910–1.898)	(0.905–2.267)
Two time			1.592∗	2.057∗∗∗
			(1.060–2.393)	(1.334–3.172)
Three times or more			1.041	1.222
			(0.676–1.602)	(0.762–1.959)
**SRH before floating**				
Favorable				1.000
Not favorable				2.282∗∗∗
				(1.404–3.708)
**SRH after floating**				
Favorable				1.000
Not favorable				6.361∗∗∗
				(4.591–8.813)
**Constant**	2.592	1.307	0.740	0.163
	(1.864–3.604)	(0.649–2.634)	(0.335–1.637)	(0.0626–0.426)
**Pseudo-R**^**2**^	0.0248	0.0616	0.0686	0.1642
**Observations**	1058	1058	1058	1058

a: Unadjusted crude model; b:Multivariate model adjusted for demographic and socioeconomic characteristics, as well as years of residency; c:Multivariate model adjusted for the frequency of physical examinations in the past year, the frequency of physical examinations in the past year based on model 2; d:Multivariate model adjusted for SRH before floating and SRH after floating based on Model 3; SRH:self-rated health; UEBMI: urban employee basic medical insurance; URRBMI: urban and rural residents basic medical insurance; FI:free insurance; CMI: Commercial medical insurance. OR:odds ratio; CI: Confidence Interval; ∗p < 0.05; ∗∗p < 0.01; ∗∗∗p < 0.001.

### 3.4 Model validation

**Fig. 1:**
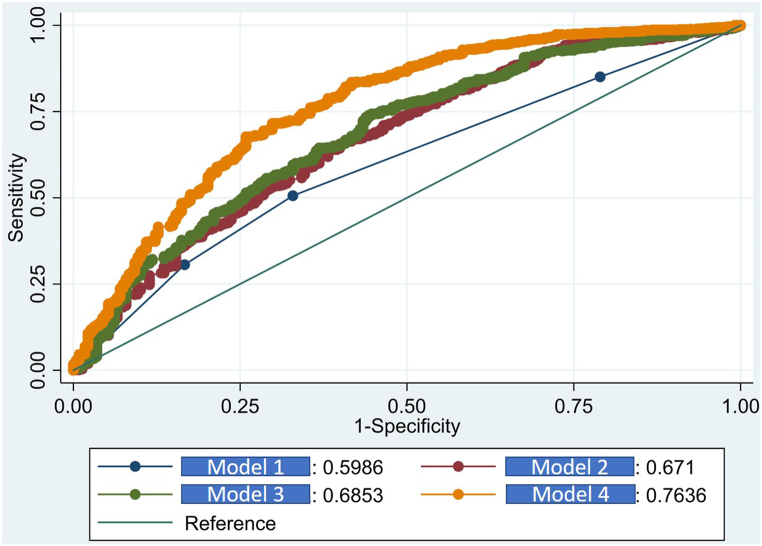
Comparisons of ROCs for model 1, 2, 3 and 4.

Fig. 1 reveals that Model 4 had the highest AUC among the four models (Model 1: 0.5986; Model 2: 0.671; Model 3: 0.686; and Model 4: 0.764), indicating its excellent predictive ability. For model fitting, the AIC indices of Models 1, 2, 3, and 4 were 1133.64, 1114.86, 1089.97, and 987.74, respectively. As a result, Model 4 was chosen as the final model. (data not shown).

## 4 Discussion

### 4.1 Main points of the study

The majority of research utilizing the segmented assimilation theory has primarily concentrated on diet-related diseases or health outcomes resulting from international migration, and few studies have applied this theory to investigate physician-patient relationships, particularly physician trust, a critical topic in China's megacities. The present study applied the segmented assimilation theory to expand research on physician-patient relationships in the context of internal migration within China. The results from this study offer preliminary insights for comprehending how internal migrants in China exhibit the four patterns posited by the segmented assimilation theory, and physician trust was linked to varying assimilation patterns among internal migrants.

#### 4.1.1 Four patterns of segmented assimilation

This study identifies four patterns of segmented assimilation: first-generation classic assimilation, first-generation integrative assimilation, first-generation segmentation, and second-generation underclass assimilation. This finding is consistent with previous literature, supporting H1 [[22], [23], [24], [25]]. This indicates the generalization of the segmented assimilation theory to both immigrants and internal migrants. When it comes to international immigration or internal migration within a country, migrants face challenges such as assimilation, acculturation, and establishing trust with healthcare providers when entering a new place and utilizing the healthcare system. According to the segmented assimilation theory, different assimilation patterns may occur due to a variety of factors, such as cultural adaptability, education, and income attainment.

#### 4.1.2 The first-generation classic and integrative assimilation and physician trust

The study revealed that respondents in the first-generation classic and integrative assimilation groups were more inclined to express a high degree of trust in physicians compared to those in the first-generation segmentation group, who retained various aspects of their regional identity. These findings support hypotheses H2a and H2b. In contrast, being a second-generation underclass-assimilated migrant had no salient effect on physicians’ trust, thus rejecting H2c. This result may have been obtained because, relative to respondents in the segmented assimilation group, those in the classic and integrative assimilation groups achieved high levels of education and income after floating, gradually improved their local cultural adaptability (including language proficiency and use), and assimilated into local economies and cultural environments, thereby enabling them to adapt to local health systems and enhancing their confidence in the local healthcare system and the trust in physicians grew [45,46]. These results align with the findings from other research [17,[46], [47], [48], [49], [50], [51], [52], [53]]. In Shanghai, migrants with a higher proficiency in Shanghainese perceiving themselves as more aligned with their physicians regarding beliefs, values, and linguistic proficiency, which led to stronger physician-patient relationships. Numerous researchers, such as Street et al., Hsueh et al., and Lor et al., posited that greater perceived personal similarity correlates with increased trust, satisfaction, and adherence [53,54].

Notably, in the present study, respondents in the first-generation integrative assimilation group reported the highest level of physician trust among the three segmented assimilation groups. This finding can be explained using Berry's acculturation coping strategy theory. According to his theory, migrants in an integrative assimilation group are regarded as bicultural migrants who are more inclined to receive support and information that facilitates their access to superior medical services from both their host and home cities. In contrast, other migrants may only receive support from a particular community or might struggle to find any help at all [14]. Conversely, although migrants in the first-generation segmentation groups have a better standing in terms of income and education compared to other groups, they cannot achieve social and cultural integration, leading to poor host city adaptation and the absence of a sense of belonging to their host cities, which further reduces their trust in the medical and health systems and physicians of their host cities. In addition, institutional factors (e.g., household registration system [46] and social exclusion [48]) and non-institutional factors (e.g., lack of community integration and support from social capital [49]) can also influence the psychological state of migrants in these groups, leading to decreased physician trust. These results align with the findings of other research as well [17,55].

#### 4.1.3 Other factors influencing physician trust

It was implied from this study that undergraduate and postgraduate education attainment, an annual income of <400 000 yuan, visiting the physician ≥2 times in the past year, and SRH status before and after population floating were all significantly correlated with trust in physicians, which aligns with the results of other research [44] [1,18,51,[56], [57], [58], [59], [60], [61]]. However, concerning the previously mentioned finding that SRH affects physician trust, some researchers argue that it has a negative impact on SRH. This is because a decreased level of interpersonal trust in physicians leads to lower health service utilization, delays in disease diagnosis and treatment, and ultimately contributes to poorer self-reported health status [62]. Therefore, further clarification is needed regarding the relationship between SRH and trust in physicians.

### 4.2 Advantages and disadvantages of the study

The study's strengths can be encapsulated as follows:

First, the present study applied the theory of segmentation assimilation to expand research on physician-patient relationships in the context of internal migration within China. It contributes theoretically to the literature on acculturation and physician-patient relationships by clarifying the mechanism by which acculturation affects physician trust among internal migrants through segmented acculturation assimilation patterns, thereby providing insights into internal migration within China and other developing countries.

Second, this study focuses on internal migrants and contributes to the enhancement of research on different subgroups within the immigrant population in developing countries such as China. Currently, medical resources in China are predominantly concentrated in major cities, particularly first-tier cities such as Beijing, Shanghai, Guangzhou, and Shenzhen. Among these internal migrants, many from the areas surrounding these cities seek treatment at tertiary hospitals for serious and complex diseases. A special group of medical immigrants has emerged and medical immigration has gradually become a common occurrence in China. Given that regional disparities in medical resources are common worldwide, this study, which focuses on internal migrants, is beneficial for other countries grappling with healthcare resource allocation challenges related to a group of medical immigrants.

The current research has various limitations that need to be recognized in order to prevent overinterpreting our findings. First, the study sample mostly comprised young migrants with an average age of <40 years (85.80 %). The skewed distribution of data and family structures affected the division patterns of the segmented assimilation process, social and economic resource acquisition, and individual acculturation. For example, all else being equal, the income of a two-parent family is higher than that of a single-parent family. Moreover, acculturation can vary among different groups of intergenerational migrants (i.e., second-vs. third-generation migrants). In this study, we lacked a third-generation group because of the age structure of the participants. Future studies should comprehensively examine the family structure and acculturation heterogeneity among internal migrants to clarify the segmentation assimilation of physician trust. Second, the dependent variable in the present study was self-reported, which could have led to recall bias. Third, due to data availability constraints, we did not consider the impact of personality variables, as extroversion may also influence trust in others, leading to omitted variables. Fourth, physicians' trust and health status, calculated based on the scales, were continuous variables. Defining these variables as binary variables based on simple means may result in the loss of information contained in the data. Using multiple categories is generally preferable for dichotomizing further studies. Finally, the present study used cross-sectional data and mainly surveyed white-collar workers (65.86 %); thus, future studies should expand the scope of this survey with longitudinal data to study the segmented assimilation trajectories of physician trust among internal migrants, thereby increasing the generalizability of the present study's findings.

## 5 Conclusion and recommendation

In summary, the present study used data on internal migrants in Shanghai, China, to examine the segmented assimilation trajectories of physician trust among internal migrants, and the results supported the theory of segmented assimilation. The internal migrants studied exhibited four patterns, as posited by the theory of segmented assimilation. Moreover, compared with the first-generation segmented assimilation groups, migrants in the first-generation classic and integrative assimilation groups were more inclined to express a high degree of trust in their physicians. Additionally, other factors that significantly influenced trust in physicians among internal migrants in China were identified, such as educational attainment, annual income, physician visits in the past year, and SRH status before and after population floating.

Practically, this study's findings regarding the segmentation assimilation trajectories of physician trust among internal migrants have significant implications for improving doctor-patient relationships and migrant population governance in China's megacities. Therefore, mixed public policies from both the healthcare sector and public governors must be planned and implemented. Effective migration policies need to address the key aspect of cultural integration and help enhance the confidence of physicians when providing health services to internal migrants in megacities. Interventions designed to promote cultural assimilation (e.g., dialect courses, interventions to address inadequate access to healthcare resources, civic and cultural activities to promote interaction between internal migrants and locals, and educational programs) could significantly enhance internal migrants' trust in physicians. Accordingly, the government can formulate specific assimilation-based migrant administration projects to maintain or promote physician trust among internal migrant populations. For example, on the one hand, for internal migrants in the classic or integrative first-generation assimilation groups, their high level of trust in physicians can be maintained by ensuring that they can retain their economic and educational advantages, as well as their ability to use the local language and adapt culturally in areas such as diet, media use, and customs, achieved through the acculturation assimilation policies of the public governors. However, for internal migrants in the first-generation segmented assimilation group, the government should implement policies to enhance their cultural adaptability and improve cultural trust. This, in turn, enhances internal migrants' understanding and utilization of the healthcare system and improves physician trust. Therefore, measures and strategies should be implemented to facilitate cultural assimilation. These may include the establishment of Mandarin or Shanghai dialect training courses or education programs for this specific migrant group, organizing local cultural communication activities or education to promote cultural interactions between internal migrants and locals, and improving access to health services. Through these initiatives of voluntary assimilation, they could adapt to the everyday practices of the dominant culture in Shanghai and assimilate into Shanghai's Haipai culture, which has been influenced by the Western influx since the mid-19th century, as well as into employment communities. During cultural assimilation, side effects such as depression, loss of identity, homesickness, mental illness, cultural bereavement, and perceived social discrimination may occur among internal migrants. It is important to fully consider the preservation and maintenance of migrants' internal home culture, empower their cultural identity, and promote organization and participation in community cultural activities related to their home cultures. Third, the government could involve community administrators and primary healthcare providers in implementing health education and promotion programs to ensure that migrants undergo regular screening for chronic diseases and achieve an improved self-reported health status.

### Ethical statement

This study was conducted in compliance with the Shanghai University of Medicine and Health Sciences Institutional Review Board for the Protection of Human Subjects on March 5, 2019 (No. 2019-gskyb-02-372424198012222511). All the participants provided written informed consent. All procedures performed in studies involving human participants followed the ethical standards with the 1964 Helsinki declaration and its later amendments or comparable ethical standards.

## Funding

This research was funded by 10.13039/501100012456National Social Science Foundation of China General Project (Grant No. 19BGL246); 10.13039/501100012456National Social Science Foundation of China Major Project (Grant No. 18ZDA088). The funders had no role in the research design, analysis or interpretation.

## Data availability statement

Data will be made available on request.

## CRediT authorship contribution statement

**Enhong Dong:** Writing – review & editing, Supervision, Resources, Project administration, Funding acquisition, Conceptualization. **Ting Xu:** Writing – original draft, Investigation, Formal analysis. **Yue Yan:** Writing – original draft. **Sheng Ji:** Writing – review & editing. **Jiahua Shi:** Investigation, Formal analysis, Data curation. **Haiwang Zhou:** Validation, Resources, Methodology, Conceptualization. **Yuping Liu:** Methodology, Writing – review & editing. **Cheng Huang:** Writing – review & editing, Validation, Resources, Methodology, Conceptualization. **Baoshan Bu:** Writing – review & editing, Supervision, Project administration, Investigation, Data curation, Conceptualization.

## Declaration of competing interest

The authors declare that they have no known competing financial interests or personal relationships that could have appeared to influence the work reported in this paper.
