# Supplementary material for: Segmented assimilation trajectories of physician trust among internal migrants in Shanghai, China: A cross-sectional study
Source: Heliyon. 2024 Sep 18;10(19):e37833. doi: 10.1016/j.heliyon.2024.e37833 (PMC11472076; doi:10.1016/j.heliyon.2024.e37833)
Supplement: Multimedia component 1 [file mmc1.docx]

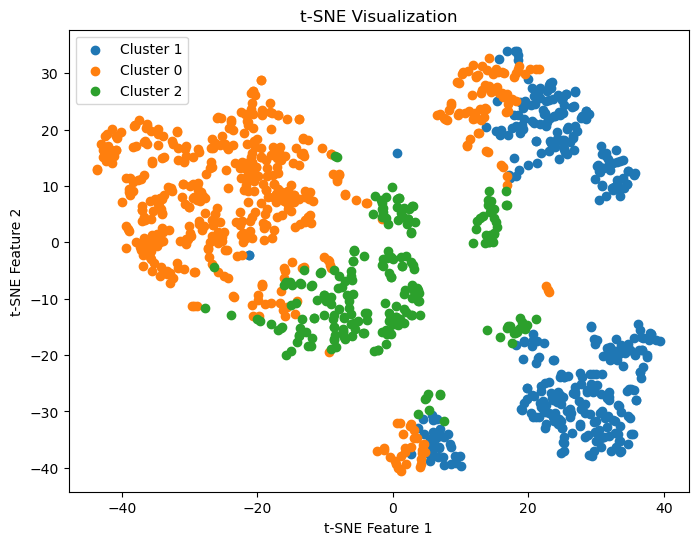


**Figure S1 T-SNE Visualization of Cluster Analysis With Three Main Patterns of Assimilation**


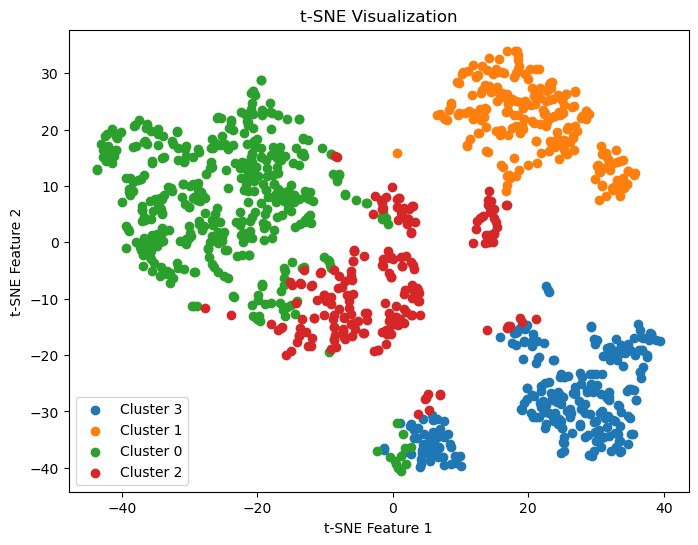


**Figure S2 T-SNE Visualization of Cluster Analysis With Four Main Patterns of Assimilation**


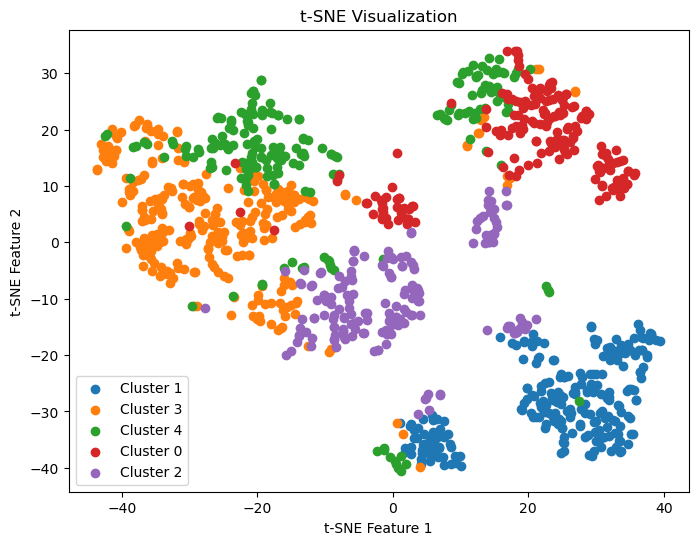


**Figure S3 T-SNE Visualization of Cluster Analysis With Five Main Patterns of Assimilation**
