# Supplementary material for: Segmented assimilation trajectories of physician trust among internal migrants in Shanghai, China: A cross-sectional study
Source: Heliyon. 2024 Sep 18;10(19):e37833. doi: 10.1016/j.heliyon.2024.e37833 (PMC11472076; doi:10.1016/j.heliyon.2024.e37833)
Supplement: Multimedia component 2 [file mmc2.docx]

**Table S1 The classification and measures of scoring variables**

| **Variables** | **How to measure** | **Type** |
| --- | --- | --- |
| Physician trust | 1=Good；0= Bad | Categorical |
| **Years of residency** | 1= 6 months-1 year;2= 1–5 years; 3=5–10 years;4= ≥ 10 years | Categorical |
| **Age** | 1=≤39 old years; 2=≥40-60 old years | Categorical |
| **Gender** | 1=Male; 0=Female | Categorical |
| **Marital status** | 1=Yes ;0=No | Categorical |
| **Educational level** | 1=High school or below;2= University (including junior college);3 =Graduate or above | Categorical |
| **Professional type** | 1=Unemployed;2=Civil sevant;3= Company or enterprise;4=Migtant workers;5= Skilled worker | Categorical |
| **Place of birth^a^** | 1=Shanghai; 2=Eastern China(excluding Shanghai);3=Central china;4=Western China; 4= Northeastern China | Categorical |
| **Insured type** | 1＝UEBMI; 2=URRBMI; 3=FI; 4=CMI or others | Categorical |
| **Annual income** | 1= CNY100,000 or below ;2= CNY110,000 -CNY250,000;3= CNY260,000 -CNY400,000;4= CNY410,000 -CNY600,000; 5= CNY600,000 or more | Categorical |
| **The frequency of physical examinations in the past year** | 1=None;2=once;3=Two times;4=Three times or more | Categorical |
| **The frequency of physician visits physician in the past year** | 1=None;2=once;3=Two times;4=Three times or more | Categorical |
| **The preferred health-care institution** | 1=Hospitials; 2=Primary care institutions | Categorical |
| **The number of disease І you have** | 0= None of the above;2=One；2=Two or more | Categorical |
| **The number of disease Ⅱ you have** | 0= None of the above;1=One or more | Categorical |
| **SRH** | 1=Favorable ；0= Not favorable | Categorical |

a: Eastern region includes 10 provinces (municipalities): Beijing, Tianjin, Hebei, Liaoning, Shanghai, Jiangsu, Zhejiang, Fujian, Shandong, Guangdong and Hainan; Central region includes 6 provinces: Shanxi, Anhui, Jiangxi, Henan, Hubei and Hunan; Western region includes 12 provinces (autonomous regions, municipalities): Inner Mongolia, Guangxi, Chongqing, Sichuan, Guizhou, Yunnan, Tibet, Shaanxi, Gansu, Qinghai, Ningxia and Xinjiang; North east China (also known as Inner Manchuria) includes Heilongjiang, Jilin and Liaoning. UEBMI: urban employee basic medical insurance ; URRBMI: urban and rural residents basic medical insurance; FI:free insurance; CMI: Commercial medical insurance; SRH:self-rated health.

**Table S2 Mean and Standard deviation (in parentheses) of four patterns of segmented assimilation （n=1111）**

| **Variables** | **First-generation classic assimilation and (n=210)** | **First-generation integrative assimilation**  **(n=301)** | **First-generation segmentation**  **(n=176)** | **Second-generation underclass assimilation**  **(n=424)** |
| --- | --- | --- | --- | --- |
| Education level | 3.03(0.74) | 3.04(0.61) | 2.90(0.63) | 2.57(0.86) |
| Annual income | 2.38(0.96) | 2.23(0.88) | 2.22(0.91) | 1.88(0.93) |
| Acculturation | 9.54(3.84) | 9.68(2.98) | 10.11(3.15) | 9.62(2.77) |
| The adaption of one’s host culture | 13.79(3.95） | 16.26(3.50) | 29.01(4.74) | 24.57(3.02) |
| The maintenance of one’s origin culture | 17.35（4.16） | 29.07(3.96) | 31.83(3.63） | 22.96(3.44) |
| Proficiency of listening local dialect | 0.86(0.35) | 0.78(0.41) | 0.65(0.0.48) | 0.520.50) |
| Proficiency of speaking local dialect | 0.68(0.47) | 0.67(0.47) | 0.50（0.50） | 0.70(0.46) |
