# Supplementary material for: Segmented assimilation trajectories of physician trust among internal migrants in Shanghai, China: A cross-sectional study
Source: Heliyon. 2024 Sep 18;10(19):e37833. doi: 10.1016/j.heliyon.2024.e37833 (PMC11472076; doi:10.1016/j.heliyon.2024.e37833)
Supplement: Multimedia component 3 [file mmc3.pdf]

The full questionaire

Dear,

In order to respond to the national requirements for the new medical reform and the population management of mega-cities, improve the medical experience and urban adaptability of the internal migrant population in our city, and enhance the soft power of Shanghai, the project research team is conducting a survey on the cultural adaptation, doctor-patient relationship and health status among internal migrants seeking medical treatment in this city based on the National Social Science Fund project "A Study on Segmented Assimilation Trajectories of Doctor-Patient Relationship among internal migrants and Its Influence Path in mega-cities of Mainland China" (No.: 19BGL246). Therefore, we sincerely request for you to fill in this questionaire according to your actual experience and condition. Thanks!

National Social Science Fund Project Team of Shanghai Jiao Tong University

July 2021

Note: The internal migrants here only refers to the agricultural peasants flowing from rural areas to urban cities or the non-agricultural residents flowing from urban cities to urban cities.

1. Personal situation

The time when you started to settle in this city (more than 6 months): [Single-choice question] *

○Below 6 months (please skip to the end of the questionnaire and submit the answer sheet)

○ Half a year - 1 year

○1-5 years

○6-10 years

○More than 10 years

Your age is: [Single choice question] *

○Under the age of 18 ○18~39 years old ○40~60 years old ○Over 60 years old

Your gender: [Single-choice question] *

○Male	○ female	○Non-heterosexuals

Your height is: cm [fill in the blank] *

____

Your weight is: kg [fill in the blank] *

____

What is your marital status: [Single choice question] *

○Single

○Married

○Divorced

○Widowed

Your parents’ place of birth: [Single-choice question] *

○Both parents were born in other places

○One parent was born in other places, and the other parent was born in the local area.

○Both parents were born locally

○Others

Area where you currently live: [Single choice question] *

○Huangpu District	○Xuhui District	○Changning District	○Jing'an District
○Putuo District	○Hongkou District	○Yangpu District	○Minhang District
○Baoshan District	○Jiading District	○Pudong New Area	○Jinshan District
○Songjiang District	○Qingpu District	○Fengxian District	○Chongming District

Your education level: [Single choice question] *

○Elementary school and below

○Junior high school

○High school (including secondary vocational school)

○University (including junior college)

○Graduate students and above

Your current career status: [Single choice question] *

○No job (unemployed and at home)

○Government civil servants or staff of agencies and institutions

○Company (enterprise) management, business, service and other white-collar personnel

○Migrant migrant workers

○Professional and technical personnel with advanced professional qualification certificates

○Professional technicians with intermediate or primary vocational qualification certificate

○Retired

○Others

Your current medical insurance situation: [Single choice question] *

○Urban employee medical insurance

○Medical insurance for urban and rural residents

○Public medical care

○Commercial medical insurance

○Long-term care insurance

○Other medical insurance

○No insurance

Before entering this city, where did you live for a long time? [Single-choice question] *

○Shanghai City (please skip to question 15)

○Eastern region (Beijing, Tianjin, Hebei, Jiangsu, Zhejiang, Fujian, Shandong, Guangdong and Hainan)

○Central region (Shanxi, Anhui, Jiangxi, Henan, Hubei and Hunan)

○Western region (Inner Mongolia, Guangxi, Chongqing, Sichuan, Guizhou, Yunnan, Tibet, Shaanxi, Gansu, Qinghai, Ningxia and Xinjiang)

○Northeast region (Liaoning, Jilin and Heilongjiang)

○Overseas or China's Hong Kong, Macao and Taiwan regions

Have you obtained local household registration: [Single choice question] *

○Yes (please skip to question 15)

○ no

Do you have a local residence permit (temporary residence permit): [Single choice question] *

○ yes

○ no

Your annual income before tax is: [Single choice question] *

○Below Yuan 100,000

○ Yuan 110,000-250,000

○ Yuan 260,000-400,000

○ Yuan 410,000-600,000

○ Yuan More than 600,000

The number of times you have participated in health care examinations (except for mandatory entry examinations) in the past year: [Single-choice question] *

○No

○1 time

○2 times

○3 times and above

The number of times you visited a doctor in the past year: [Single-choice question] *

○Not at all (please skip to question 19)

○1 time

○2 times

○3 times and above

If you have been to see a doctor, then the type of medical institution you have been to for the longest time is: [Single-choice question] *

○Hospitals (such as general hospitals, traditional Chinese medicine hospitals, combined Chinese and Western hospitals, specialized hospitals, nursing homes, etc.)

○Primary medical institutions (such as urban community health service centers)

○Professional public health institutions (such as maternal and child health hospitals and other specialized disease hospitals)

○Other health facilities (such as nursing homes)

2. Cultural adaptability

How well do you understand the local dialect: [Single choice question] *

○ very good ○ better ○ General ○ not good ○ very bad

How well do you speak the local dialect: [Single choice question] *

○ very good ○Relatively good ○General ○Not good ○Very bad

The language you use at home: [Single-choice question] *

○Shanghai dialect

○Mandarin

○Hometown dialect

○Foreign language

The language you use at work: [Single-choice question] *

○Shanghai dialect

○Mandarin

○Hometown dialect

○Foreign language

The language you use when socializing: [Single-choice question] *

○Shanghai dialect

○Mandarin

○Hometown dialect

○Foreign language

Do you often like to watch (listen to) programs from your hometown when you watch TV or listen to the radio at home? [Single-choice question] *

○Yes

○No

Type of friends you tend to make: [Single choice question] *

○It doesn’t matter

○Shanghai (local) people

○Fellows from this province

○People from other provinces

Changes in your diet since you came to this city: [Single-choice questions] *

○No change at all

○Some changes

○General

○Changes a lot

○Complete change

How has your communication language changed since coming to this city: [Single-choice question] *

○No change at all

○Some changes

○ General

○Various changes

○ complete change

What is the change in media usage (newspapers, magazines, TV, radio, Internet, mobile phones and other electronic products, etc.) since you came to this city: [Single-choice question] *

○No change at all

○Some changes

○General

○Changes a lot

○Complete change

Changes in customs (such as weddings, burials, etc.) after you came to this city: [Single-choice question] *

○No change at all

○Some changes

○General

○Changes a lot

○Complete change

The following questions reflect your attitude towards the local culture and the culture of your hometown. Please choose from small to large according to the actual situation.

The health habits I maintain are different from those of locals [single choice question] *

○Strongly disagree	○Disagree	○General	○Agree	○Strongly agree

I dress differently from the locals [single choice question] *

○Strongly disagree	○Disagree	○General	○Agree	○Strongly agree

My children's education philosophy is different from the locals [single choice question] *

○ strongly disagree	○Disagree	○ General	○ Agree	○ strongly agree

My perspective on the problem of doctor-patient conflict is different from that of local people [single-choice question] *

○ strongly disagree	○ disagree	○ General	○Agree	○ strongly agree

I think it is very important to abide by the customs of my hometown (such as wedding and burial customs) [Single-choice question] *

○Strongly disagree	○Disagree	○General	○Agree	○Strongly agree

I think it is very important to follow the way things are done in my hometown [single choice question] *

○ strongly disagree	○Disagree	○ General	○ Agree	○ strongly agree

I think my child should learn to speak the dialect of my hometown [single choice question] *

○Strongly disagree	○Disagree	○General	○Agree	○Strongly agree

I think it is very important to maintain the living habits (such as diet) in my hometown [single-choice question] *

○ strongly disagree	○ disagree	○ General	○ Agree	○ strongly agree

3. Doctor-patient relationship

The following questions reflect your trust in medical services. Please indicate your degree of agreement with the following statements in the space provided based on your local medical experience. Only one item can be selected for each row.

I feel that the facilities, medical staff appearance and service environment of the medical institution are all good [single-choice question] *

○ strongly agree	○ Agree	○ General	○Disagree	○Strongly disagree

I feel that the waiting time for appointment service and consultation service is very long* [Single-choice question] *

○ strongly agree	○ Agree	○ General	○ disagree	○ strongly disagree

No matter whether your condition is mild or severe, the doctor can always pinpoint the problem accurately [Single-choice question] *

○ strongly agree	○Agree	○General	○Disagree	○Strongly disagree

I am confident that the doctor's diagnosis is correct [single-choice question] *

○Strongly agree	○Agree	○General	○Disagree	○Strongly disagree

The doctor thinks more about my health than the cost of treatment [single-choice question] *

○Strongly agree	○Agree	○ General	○ disagree	○ strongly disagree

My doctor puts my interests first, not his own or the hospital's interests [Single choice question] *

○Strongly agree	○Agree	○General	○Disagree	○Strongly disagree

The dosage and time of medicine given by doctors are appropriate [single-choice question] *

○Strongly agree	○ Agree	○ General	○ disagree	○ strongly disagree

Doctors sometimes perform too many examinations and tests* [Single choice question] *

○ strongly agree	○ Agree	○General	○Disagree	○Strongly disagree

Doctors care about patients and listen to patients [single-choice questions] *

○Strongly agree	○Agree	○General	○Disagree	○Strongly disagree

The doctor will provide me with the opportunity to ask him about my condition [single choice question] *

○Strongly agree	○Agree	○General	○Disagree	○Strongly disagree

I trust the technical ability of the hospital doctors [single-choice question] *

○Strongly agree	○Agree	○General	○Disagree	○Strongly disagree

I trust the hospital in general [single choice question] *

○ strongly agree	○ Agree	○ General	○ disagree	○ strongly disagree

Combined with your past medical experience in the local area, how satisfied are you with the current medical service: [Single-choice question] *

○ very satisfied	○ Satisfied	○ General	○ Dissatisfied	○ Very dissatisfied

What do you think is the gap between the actual quality of medical services currently received and the expected quality of medical services: [Single-choice question] *

○ There is no gap	○ Little difference	○ General	○The gap is relatively large	○The gap is very large

Based on your experience of seeing a doctor or your current knowledge, what do you think is the current relationship between doctors and patients in Shanghai? [Single-choice question] *

○Very good	○Relatively good	○General	○Relatively bad	○Very bad

What is your main source of information for making the above relationship judgments? [Single-choice question] *

○Newspapers and magazines

○ Radio, radio or television

○Network

○Personal experience

○Experiences of relatives or friends around you

○Others

4. Self-reported health status

Based on your current actual health status, please conduct a self-assessment of your health status in the following options.

Before you came here, what was your evaluation of your overall health level: [Single-choice question] *

○ very good	○ better	○ General	○ relatively poor	○ very bad

Compared with before coming here, how do you feel about the indicators of physical examination: [Single-choice question] *

○ very good	○ better	○ General	○ relatively poor	○ very bad

Compared with before coming here, do you currently feel that your emotional relationship is coordinated: [Single-choice question] *

○ very good	○ better	○General	○Relatively bad	○Very bad

Compared with before coming here, do you currently feel that your family relationship is harmonious: [Single-choice question] *

○Very good ○Relatively good ○General ○Relatively bad ○Very bad

Compared with before coming here, do you currently feel that the relationship between friends is harmonious: [Single-choice question] *

○Very good ○Relatively good ○General ○Relatively bad ○ very bad

Compared with before coming here, how much social support do you currently feel: [single-choice question] *

○ very good ○ better ○ General ○ relatively poor ○ very bad

Based on your current physical condition, how many of the following diseases do you suffer from (asthma, back pain, high blood pressure, hyperlipidemia, diabetes, allergies, migraines, ulcers, bronchitis, arthritis): [Single-choice question] *

○ None of the above

○1 type

○2 types and above

According to your current physical condition, how many of the following diseases (heart disease, cancer, blood disease) do you suffer from: [Single-choice question] *

○None of the above

○1 type

○2 types and above

The questionnaire ends here, thank you for your participation. This questionnaire adopts an anonymous survey method, and the content involved in you **is strictly confidential** , so please rest assured. If you are interested in the results of this research, please leave

your contact information here, and we will send you the final research conclusions, hoping to be helpful to you. Thank you again for your cooperation! !
