# Supplementary material for: Segmented assimilation trajectories of physician trust among internal migrants in Shanghai, China: A cross-sectional study
Source: Heliyon. 2024 Sep 18;10(19):e37833. doi: 10.1016/j.heliyon.2024.e37833 (PMC11472076; doi:10.1016/j.heliyon.2024.e37833)
Supplement: Multimedia component 4 [file mmc4.docx]

**上海外来人口文化适应、医患关系及健康情况调研问卷（沪版）**

您好！
为了响应国家对新医改和超大城市人口治理的要求，改善我市外来人口的就医体验和城市适应程度，提升上海城市软实力，项目研究团队基于国家社科基金课题项目“基于SA理论超大城市外来人口医患关系分隔同化性及其影响路径研究”(编号：19BGL246)，进行本市就医市民的文化适应、医患关系和健康状况调查，请您根据自己的实际情况进行填写。谢谢！

上海交通大学国家社科基金项目组

2021年7月

说明：此处外来人口仅指从农村到城市的农业常住人口和从城市到城市的非农业常住人口。

**一、个人情况**

您开始来到此城市定居（6个月以上）的时间： [单选题] *

| ○6个月以下 (请跳至第问卷末尾，提交答卷) |
| --- |
| ○半年-1年 |
| ○1-5年 |
| ○6-10年 |
| ○10年以上 |

您的年龄处于： [单选题] *

| ○18岁以下 | ○18~39岁 | ○40~60岁 | ○60岁以上 |  |  |  |
| --- | --- | --- | --- | --- | --- | --- |

您的性别： [单选题] *

| ○男 | ○女 | ○非异性恋者 |  |  |  |  |  |
| --- | --- | --- | --- | --- | --- | --- | --- |

您的身高是：厘米 [填空题] *

_________________________________

您的体重是：公斤 [填空题] *

_________________________________

您婚姻状况是： [单选题] *

| ○未婚 |
| --- |
| ○已婚 |
| ○离异 |
| ○丧偶 |

您的父母出生地情况： [单选题] *

| ○父母双方都出生在外地 |
| --- |
| ○父母一方出生在外地，一方在本地 |
| ○父母都出生在本地 |
| ○其他 |

您目前居住的区域： [单选题] *

| ○黄浦区 | ○徐汇区 | ○长宁区 | ○静安区 |
| --- | --- | --- | --- |
| ○普陀区 | ○虹口区 | ○杨浦区 | ○闵行区 |
| ○宝山区 | ○嘉定区 | ○浦东新区 | ○金山区 |
| ○松江区 | ○青浦区 | ○奉贤区 | ○崇明区 |

您的文化程度： [单选题] *

| ○小学及以下 |
| --- |
| ○初中 |
| ○高中（含中职） |
| ○大学（含大专） |
| ○研究生及以上 |

您目前的职业状态： [单选题] *

| ○无职业（待业在家） |
| --- |
| ○政府公务员或者机关、事业单位人员 |
| ○公司（企业）管理、业务、办事等白领人员 |
| ○外来农民务工者 |
| ○有高级职业资格证专业技术人员 |
| ○有中级或初级职业资格证专业技术人员 |
| ○已退休 |
| ○其他 |
| ○选项290  依赖于 |
| ○选项291  依赖于 |

您目前医疗保险情况： [单选题] *

| ○城镇职工医疗保险 |
| --- |
| ○城乡居民医疗保险 |
| ○公费医疗 |
| ○商业医疗保险 |
| ○长期护理险 |
| ○其他医疗保险 |
| ○无保险 |

进入本城市前，您曾长期生活居住在哪里？ [单选题] *

| ○上海市 (请跳至第15题) |
| --- |
| ○东部地区（北京、天津、河北、江苏、浙江、福建、山东、广东和海南） |
| ○中部地区（山西、安徽、江西、河南、湖北和湖南） |
| ○西部地区（内蒙古、广西、重庆、四川、贵州、云南、西藏、陕西、甘肃、青海、宁夏和新疆） |
| ○东北地区（辽宁、吉林和黑龙江） |
| ○国外或者中国港澳台地区 |

您是否已经获得本地户籍： [单选题] *

| ○是 (请跳至第15题) |
| --- |
| ○否 |

您是否有本地居住证（暂住证）： [单选题] *

| ○是 |
| --- |
| ○否 |

您税前年收入是： [单选题] *

| ○10万以下 |
| --- |
| ○11-25万 |
| ○26-40万 |
| ○41-60万 |
| ○60万以上 |

您过去一年内参加医疗保健体检（强制性入职体检除外）的次数： [单选题] *

| ○没有 |
| --- |
| ○1次 |
| ○2次 |
| ○3次及以上 |

您过去一年内的就医次数是： [单选题] *

| ○完全没有 (请跳至第19题) |
| --- |
| ○1次 |
| ○2次 |
| ○3次及以上 |

如果曾经就医，那么您就医时间最长的医疗机构类别是： [单选题] *

| ○医院（比如综合医院、中医院、中西结合医院、专科医院、护理院等） |
| --- |
| ○基层医疗机构（比如城市社区卫生服务中心） |
| ○专业公共卫生机构（比如妇幼保健院等专科疾病医院） |
| ○其他卫生机构（比如疗养院） |

**二、文化适应性情况**

您能听懂本地方言程度： [单选题] *

| ○非常好 | ○比较好 | ○一般 | ○不好 | ○非常不好 |
| --- | --- | --- | --- | --- |

您能说本地方言程度： [单选题] *

| ○非常好 | ○比较好 | ○一般 | ○不好 | ○非常不好 |
| --- | --- | --- | --- | --- |

您在家里使用的语言情况： [单选题] *

| ○上海话 |
| --- |
| ○普通话 |
| ○家乡话 |
| ○外语 |

您在工作时使用的语言情况： [单选题] *

| ○上海话 |
| --- |
| ○普通话 |
| ○家乡话 |
| ○外语 |

您在社交时使用的语言情况： [单选题] *

| ○上海话 |
| --- |
| ○普通话 |
| ○家乡话 |
| ○外语 |

您在家看电视或听广播会经常喜欢看（听）家乡的节目吗？ [单选题] *

| ○是 |
| --- |
| ○否 |

您倾向于交朋友的类型： [单选题] *

| ○无所谓 |
| --- |
| ○上海（本地）人 |
| ○本省老乡 |
| ○其他省的人 |

您来到此城市后的饮食变化情况： [单选题] *

| ○完全没有变化 |
| --- |
| ○有点变化 |
| ○一般 |
| ○变化很大 |
| ○完全变化 |

您来到此城市后的交流语言变化情况是： [单选题] *

| ○完全没有变化 |
| --- |
| ○有点变化 |
| ○一般 |
| ○变化很大 |
| ○完全变化 |

您来到此城市后的媒体使用（报纸、杂志、电视、电台、互联网和手机等电子产品等）变化情况是： [单选题] *

| ○完全没有变化 |
| --- |
| ○有点变化 |
| ○一般 |
| ○变化很大 |
| ○完全变化 |

您来到此城市后的风俗习惯（比如婚葬嫁娶等）变化情况是： [单选题] *

| ○完全没有变化 |
| --- |
| ○有点变化 |
| ○一般 |
| ○变化很大 |
| ○完全变化 |

以下问题反映你对本地文化和家乡文化态度，请根据实际情况进行程度由小到大的选择。

我保持的健康习惯与本地人不同 [单选题] *

| ○非常不同意 | ○不同意 | ○一般 | ○同意 | ○非常同意 |
| --- | --- | --- | --- | --- |

我的穿着打扮与本地人不同 [单选题] *

| ○非常不同意 | ○不同意 | ○一般 | ○同意 | ○非常同意 |
| --- | --- | --- | --- | --- |

我的小孩教育理念与本地人不同 [单选题] *

| ○非常不同意 | ○不同意 | ○一般 | ○同意 | ○非常同意 |
| --- | --- | --- | --- | --- |

我看待医患矛盾问题的观点与本地人不同 [单选题] *

| ○非常不同意 | ○不同意 | ○一般 | ○同意 | ○非常同意 |
| --- | --- | --- | --- | --- |

我觉得遵守我家乡的习俗（比如婚葬习俗）非常重要 [单选题] *

| ○非常不同意 | ○不同意 | ○一般 | ○同意 | ○非常同意 |
| --- | --- | --- | --- | --- |

我觉得按照家乡的做事方式非常重要 [单选题] *

| ○非常不同意 | ○不同意 | ○一般 | ○同意 | ○非常同意 |
| --- | --- | --- | --- | --- |

我觉得我的孩子应该学说家乡方言 [单选题] *

| ○非常不同意 | ○不同意 | ○一般 | ○同意 | ○非常同意 |
| --- | --- | --- | --- | --- |

我觉得保持家乡的生活习惯（比如饮食）非常重要 [单选题] *

| ○非常不同意 | ○不同意 | ○一般 | ○同意 | ○非常同意 |
| --- | --- | --- | --- | --- |

**三、医患关系**
以下问题反映您对医疗服务的信任程度。请结合您在本地的就医经历，在空格内标明你对下列陈述的认同程度，每一行只能选择一项。

我感觉医疗机构的设施、医务人员仪表和服务环境都是不错的 [单选题] *

| ○非常同意 | ○同意 | ○一般 | ○不同意 | ○非常不同意 |
| --- | --- | --- | --- | --- |

我感觉等候预约服务和咨询服务的时间都很长* [单选题] *

| ○非常同意 | ○同意 | ○一般 | ○不同意 | ○非常不同意 |
| --- | --- | --- | --- | --- |

不管你的病情是轻还是重，医生总能准确地指出问题所在 [单选题] *

| ○非常同意 | ○同意 | ○一般 | ○不同意 | ○非常不同意 |
| --- | --- | --- | --- | --- |

我对医生的诊断正确充满了信心 [单选题] *

| ○非常同意 | ○同意 | ○一般 | ○不同意 | ○非常不同意 |
| --- | --- | --- | --- | --- |

医生更多地考虑我的健康而不是治疗成本 [单选题] *

| ○非常同意 | ○同意 | ○一般 | ○不同意 | ○非常不同意 |
| --- | --- | --- | --- | --- |

我的医生把我的利益放在第一位，而不是他自己或医院利益 [单选题] *

| ○非常同意 | ○同意 | ○一般 | ○不同意 | ○非常不同意 |
| --- | --- | --- | --- | --- |

医生们给的药剂量和时间是合适的 [单选题] *

| ○非常同意 | ○同意 | ○一般 | ○不同意 | ○非常不同意 |
| --- | --- | --- | --- | --- |

医生们有时会做过多的检查和化验* [单选题] *

| ○非常同意 | ○同意 | ○一般 | ○不同意 | ○非常不同意 |
| --- | --- | --- | --- | --- |

医生很关心患者，倾听患者诉说 [单选题] *

| ○非常同意 | ○同意 | ○一般 | ○不同意 | ○非常不同意 |
| --- | --- | --- | --- | --- |

医生会对我提供向他询问病情的机会 [单选题] *

| ○非常同意 | ○同意 | ○一般 | ○不同意 | ○非常不同意 |
| --- | --- | --- | --- | --- |

我对医院医生们的技术能力是信任的 [单选题] *

| ○非常同意 | ○同意 | ○一般 | ○不同意 | ○非常不同意 |
| --- | --- | --- | --- | --- |

我对医院总体是信任的 [单选题] *

| ○非常同意 | ○同意 | ○一般 | ○不同意 | ○非常不同意 |
| --- | --- | --- | --- | --- |

结合过去您在本地的就医经历，您对目前医疗服务的满意程度是： [单选题] *

| ○非常满意 | ○满意 | ○一般 | ○不满意 | ○非常不满意 |
| --- | --- | --- | --- | --- |

您觉得目前所实际接受的医疗服务质量与期望的医疗服务质量差距有多大： [单选题] *

| ○没有差距 | ○差距不大 | ○一般 | ○差距比较大 | ○差距非常大 |
| --- | --- | --- | --- | --- |

结合您的看病经历或者目前的认知情况，您认为当前在上海，医生和病人之间的关系是怎样的？ [单选题] *

| ○非常好 | ○比较好 | ○一般 | ○比较差 | ○非常差 |
| --- | --- | --- | --- | --- |

您做出以上关系判断的主要信息来源是哪里？ [单选题] *

| ○报纸、杂志 |
| --- |
| ○电台、广播或电视 |
| ○网络 |
| ○亲身经历 |
| ○周围亲戚或者朋友的经历 |
| ○其他 |

**四、自述健康状况**
针对您目前的实际健康状况，请在以下选项中进行健康情况自评。

您来本地前，对自己的健康总体水平评价是： [单选题] *

| ○非常好 | ○比较好 | ○一般 | ○比较差 | ○非常差 |
| --- | --- | --- | --- | --- |

与来本地之前相比，您目前感觉身体体检各项指标： [单选题] *

| ○非常好 | ○比较好 | ○一般 | ○比较差 | ○非常差 |
| --- | --- | --- | --- | --- |

与来本地之前相比，您目前感觉情感关系协调： [单选题] *

| ○非常好 | ○比较好 | ○一般 | ○比较差 | ○非常差 |
| --- | --- | --- | --- | --- |

与来本地之前相比，您目前感觉家庭关系协调： [单选题] *

| ○非常好 | ○比较好 | ○一般 | ○比较差 | ○非常差 |
| --- | --- | --- | --- | --- |

与来本地之前相比，您目前感觉朋友关系协调： [单选题] *

| ○非常好 | ○比较好 | ○一般 | ○比较差 | ○非常差 |
| --- | --- | --- | --- | --- |

与来本地之前相比，您目前感觉得到的社会支持： [单选题] *

| ○非常好 | ○比较好 | ○一般 | ○比较差 | ○非常差 |
| --- | --- | --- | --- | --- |

根据您目前的身体状况，您患有以下疾病种类（哮喘 、背痛 、高血压、高血脂、糖尿病、过敏、偏头痛、溃疡、支气管炎、关节炎）的数量： [单选题] *

| ○以上均没有 |
| --- |
| ○1种 |
| ○2种及以上 |

根据您目前的身体状况，您患有以下疾病种类（心脏病、癌症 、血液疾病）的数量： [单选题] *

| ○以上均没有 |
| --- |
| ○1种 |
| ○2种及以上 |

问卷至此结束，感谢您的参与。本问卷采取匿名调查方式，所涉及您的内容**严格保密**，请您完全放心。如果你对本研究的结果感兴趣，请在此留下你的联系方式，我们会将最终的研究结论发送给你，希望能够对你有所帮助。再次感谢你的合作！！
